# Supplementary material for: Content-rich biological network constructed by mining PubMed abstracts
Source: BMC Bioinformatics. 2004 Oct 8;5:147. doi: 10.1186/1471-2105-5-147 (PMC528731; doi:10.1186/1471-2105-5-147)
Supplement: Additional File 2 — The original results of the above study (non-essential files are deleted to keep the file size under the limit set by BMC bioinformatics). [file 1471-2105-5-147-S2.bz2 › chilibotAdditionalFile2/dip05/717ID10781811E18054/html/TATA_TATB.html]

 


 **TATA** and **TATB** 
  
Found 20 abstracts in PubMed, retrieved 05.  
 

 What does Google say? 
 PDF only 
| .edu only 

---

**Interactive relationship** (e.g. stimulation, inhibition, etc)

**Neutral relationship**- The three isolated, affinity tagged Tat  [ **TATA** ]  complexes comprised  **TatA** ,  **TatB**  and TatC in each case, demonstrating a strong interaction between these three subunits.  Ref: 12823967 J Mol Biol, 2003
- Surprisingly, large scale mutagenesis of the conserved amphipathic regions of  **TatA**  and  **TatB**  leads only to minor effects on Tat  [ **TATA** ]  dependent export suggesting that this particular feature is not central to the translocation mechanism.  Ref: 12606028 FEBS Lett, 2003
- Its main components are  **TatA** ,  **TatB**  and TatC, all of which are required for Tat  [ **TATA** ]  dependent export.  Ref: 12773183 Biochem Soc Trans, 2003

**Non-interactive relationship** (e.g. studied together, co-existance, homology, etc.)

- The Escherichia coli twin arginine translocase conserved residues of  **TatA**  and  **TatB**  family components involved in protein transport.  Ref: 12650927 FEBS Lett, 2003
- The first region is the interdomain hinge between the transmembrane and the amphipathic alpha helices of  **TatA**  and  **TatB**  proteins.  Ref: 12650927 FEBS Lett, 2003
- The second region is within the amphipathic helices of  **TatA**  and  **TatB** .  Ref: 12650927 FEBS Lett, 2003
- In this report we have studied the functions of conserved residues within the structurally related  **TatA**  and  **TatB**  proteins.  Ref: 12650927 FEBS Lett, 2003
